# Influenza vaccine effectiveness against A(H3N2) during the delayed 2021/22 epidemic in Canada

**DOI:** 10.2807/1560-7917.ES.2022.27.38.2200720

**Published:** 2022-09-22

**Authors:** Shinhye Kim, Erica SY Chuang, Suzana Sabaiduc, Romy Olsha, Samantha E Kaweski, Nathan Zelyas, Jonathan B Gubbay, Agatha N Jassem, Hugues Charest, Gaston De Serres, James A Dickinson, Danuta M Skowronski

**Affiliations:** 1 British Columbia Centre for Disease Control, Vancouver, Canada; 2 Public Health Ontario, Toronto, Canada; 3 Public Health Laboratory, Alberta Precision Laboratories, Edmonton, Canada; 4 University of Toronto, Toronto, Canada; 5 University of British Columbia, Vancouver, Canada; 6 Institut national de santé publique du Québec, Québec, Canada; 7 Laval University, Quebec, Canada; 8 Centre Hospitalier Universitaire de Québec, Québec, Canada; 9 University of Calgary, Calgary, Canada

**Keywords:** Influenza, A(H3N2), vaccine effectiveness, clade, test-negative design, observational study

## Abstract

Influenza virus circulation virtually ceased in Canada during the COVID-19 pandemic, re-emerging with the relaxation of restrictions in spring 2022. Using a test-negative design, the Canadian Sentinel Practitioner Surveillance Network reports 2021/22 vaccine effectiveness of 36% (95% CI: −38 to 71) against late-season illness due to influenza A(H3N2) clade 3C.2a1b.2a.2 viruses, considered antigenically distinct from the 3C.2a1b.2a.1 vaccine strain. Findings reinforce the World Health Organization’s decision to update the 2022/23 northern hemisphere vaccine to a more representative A(H3N2) clade 3C.2a1b.2a.2 strain.

In Canada, as elsewhere, public health measures such as physical distancing, masking requirements and vaccine passports were implemented early in the coronavirus disease (COVID-19) pandemic to control transmission of severe acute respiratory syndrome coronavirus 2 (SARS-CoV-2) [1]. These measures probably also interrupted circulation of other respiratory viruses, including influenza. After they were lifted across Canada at the beginning of March 2022, influenza A(H3N2) virus circulation sharply increased, surpassing the seasonal epidemic threshold in week 16 (beginning 14 April 2022) with an unusually late peak in May 2022 [2].

Using a test-negative design (TND), the Canadian Sentinel Practitioner Surveillance Network (SPSN) assessed 2021/22 vaccine effectiveness (VE) against late-season A(H3N2) illness from March to July of 2022. With the incorporation of SARS-CoV-2 surveillance into SPSN activities, we also explored confounding due to potentially correlated influenza and COVID-19 vaccination behaviours, recently suggested by others as influential on influenza VE estimates [3].

## Vaccine effectiveness evaluation

Nasal or nasopharyngeal specimens and epidemiological data included in VE analyses were collected from consenting patients presenting within 7 days of acute respiratory symptom onset to community-based clinics or COVID-19 assessment sites in the provinces of Alberta, British Columbia and Ontario. Influenza VE analyses were restricted as per usual to those with influenza-like illness (ILI) defined by acute onset of fever and cough, and at least one other symptom including sore throat, myalgia, arthralgia or prostration [4]. Fever was not a required symptom in elderly adults ≥ 65-years. Children younger than 1 year were excluded owing to vaccine eligibility considerations. Specimens were tested for influenza and SARS-CoV-2 viruses by nucleic acid amplification test (NAAT) at accredited provincial public health reference laboratories. The VE analyses were restricted to epidemiological weeks between the first and last influenza A(H3N2) case detection in 2022: from week 10 (starting 6 March 2022) to week 26 (ending 2 July 2022) (Figure).

**Figure:**
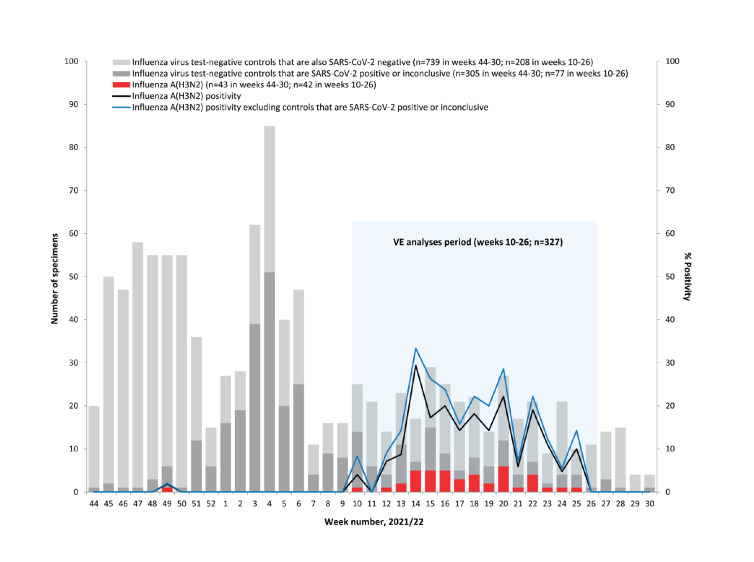
Influenza detections among eligible patients presenting with influenza-like illness by week of specimen collection, Canadian Sentinel Practitioner Surveillance Network, 1 November 2021–30 July 2022 (weeks 44–30: n = 1,087)

SARS-CoV-2: severe acute respiratory syndrome coronavirus 2; VE: vaccine effectiveness.

As per usual SPSN approach, missing specimen collection dates were imputed as the date the specimen was received and processed at the laboratory minus 2 days.

Influenza and COVID-19 vaccination status was based on participant self-report of 2021/22 vaccine receipt ≥ 2 weeks before symptom onset; patients vaccinated less than 2 weeks before symptom onset or with unknown vaccination status/timing were excluded. Virtually all of the 2021/22 influenza vaccines available in contributing Canadian provinces were egg-based and inactivated products. The 2021/22 influenza vaccine contained an A/Cambodia/e0826360/2020 (H3N2)-like virus belonging to clade 3C.2a1b.2a.1 [5].

VE was calculated as 1 − odds ratios (OR) × 100%. ORs were derived by comparing influenza test positivity between vaccinated and unvaccinated participants using logistic regression, adjusting for potential confounders as specified. In exploratory analyses we excluded COVID-19 cases (NAAT-confirmed for SARS-CoV-2) from influenza virus test-negative controls [3], and addressed relatively small sample sizes using Firth’s method of penalised logistic regression (PLR) [6-8]. 

## Epidemiological findings

Of 327 eligible specimens collected during the analysis period, 42 (13%) tested positive for influenza virus and all were A(H3N2) (Figure). Of the A(H3N2) viruses genetically characterised in Canada, all were clade 3C.2a1b.2a.2, related to the upcoming northern hemisphere 2022/23 vaccine strain [5], but considered antigenically distinct from the 2021/22 clade 3C.2a1b.2a.1 vaccine [2,9]. Participant profiles stratified by case and vaccine status are displayed in Table 1. More 9–19-years-olds contributed as cases (38%) than controls (11%) (p = 0.02), with that age group having the highest influenza test positivity (33%; 16/48). 

**Table 1:** Participant profile, including any influenza virus test-negative controls, 2021/22 influenza vaccine effectiveness evaluation, Canadian Sentinel Practitioner Surveillance Network, 6 March–2 July 2022 (n = 327)

Characteristics	All participants (column %)							Proportion influenza vaccinated^a^ (row %)						
	Overall		Influenza A(H3N2) cases		Influenza virus test-negative controls (any)^b^		p value^c^	Overall		p value^c^	Influenza A(H3N2) cases		Influenza virus test-negative controls (any)^b^	
	n	%	n	%	n	%		n	%		n	%	n	%
N (row %)	327	100	42	13	285	87	NA	170	52	NA	15	36	155	54
**Age group (years)**														
1–8	62	19	6	14	56	20	< 0.001	20	32	< 0.001	2	33	18	32
9–19	48	15	16	38	32	11		18	38		3	19	15	47
20–49	125	38	13	31	112	39		59	47		5	38	54	48
50–64	51	16	3	7	48	17		37	73		1	33	36	75
≥ 65	41	13	4	10	37	13		36	88		4	100	32	86
**Median (range)**	34 (1–95)		18.5 (2–81)		36 (1–95)		0.033	43.5 (1–95)		< 0.001	38 (2–81)		44 (1–95)	
**Interquartile range**	13–53		13–38		15–55		NA	28–62		NA	18–66		29–62	
**Sex**														
Female	195	60	22	52	173	61	0.280	107	55	0.204	9	41	98	57
Male	130	40	20	48	110	39		62	48		6	30	56	51
Unknown	2	1	0	0	2	1	NA	1	50	NA	0	0	1	50
**Comorbidity^d^**														
No	227	69	36	86	191	67	0.014	99	44	< 0.001	11	31	88	46
Yes	100	31	6	14	94	33		71	71		4	67	67	71
**Province**														
Alberta	150	46	25	60	125	44	0.154	70	47	0.086	6	24	64	51
British Columbia	75	23	8	19	67	24		38	51		2	25	36	54
Ontario	102	31	9	21	93	33		62	61		7	78	55	59
**Specimen collection interval from onset of influenza-like illness^e^**														
≤ 4 days	242	74	35	83	207	73	0.140	127	52	0.764	12	34	115	56
5–7 days	85	26	7	17	78	27		43	51		3	43	40	51
**Median**	3		2.5		3		0.048	3		0.274	3		3	
**Month of specimen collection, 2022**														
March	78	24	4	10	74	26	0.029	43	55	0.079	1	25	42	57
April	97	30	18	43	79	28		40	41		4	22	36	46
May	92	28	15	36	77	27		51	55		8	53	43	56
June–2 July	60	18	5	12	55	19		36	60		2	40	34	62

NA: not applicable.

Unless otherwise specified, values displayed in the columns represent the number of specimens per category and percentages are relative to the total.

^a^ Vaccination status based on patients’ self-report; defined as receipt of 2021/22 seasonal influenza vaccine at least 2 weeks before symptom onset. Patients vaccinated less than 2 weeks before onset of symptoms or with unknown vaccination status or timing were excluded.

^b^ Influenza virus test-negative specimens, including those that tested positive (n=76) or inconclusive (n=1) for SARS-CoV-2 by nucleic acid amplification test. For participant distributions excluding these 77 specimens from influenza virus test-negative controls, see Supplementary Table S1.

^c^ p values for comparison between cases and controls or for the proportion vaccinated were derived by chi-squared test or Wilcoxon rank-sum test.

^d^ Includes chronic comorbidities that place individuals at higher risk of serious complications from influenza as defined by Canada’s National Advisory Committee on Immunization, including: heart, pulmonary (including asthma), renal, metabolic (such as diabetes), blood, cancer or immunocompromising conditions, conditions that compromise management of respiratory secretions and increase risk of aspiration, or morbid obesity (body mass index ≥ 40).

^e^ As per usual SPSN approach, missing specimen collection dates were imputed as the date the specimen was received and processed at the laboratory minus 2 days.

In exploratory analyses excluding the 77 (27%) of 285 influenza virus test-negative controls that were NAAT positive (n = 76) or inconclusive (n = 1) for SARS-CoV-2, participant characteristics were similar (for the detailed results on this see Supplementary Table S1). Of these 77 participants, 72 (93%) were vaccinated against COVID-19. Compared with the SARS-CoV-2-negative controls shown in Supplementary Table S1, SARS-CoV-2-positive and inconclusive controls were older (median: 30 and 45 years, respectively) (p<0.001), more often with comorbidity (62/208 (30%) and 32/77 (42%), respectively) (p = 0.061), and vaccinated against influenza (108/208 (52%) and 47/77 (61%), respectively) (p = 0.170).

Overall, 54% of negative controls and 36% of influenza cases were vaccinated (p = 0.024) (Table 1). Age and comorbidity were identified as confounders with significant variation (p < 0.05) based both upon percentage test-positive and vaccinated. Neither test positivity nor vaccination were statistically different between provinces when SARS-CoV-2-positive or inconclusive specimens were included among influenza test-negative controls (Table 1), but both were significant (p < 0.05) when such specimens were excluded (for these exploratory analyses see Supplementary Table S1). Variation by month was significant for the likelihood of test positivity but not for being vaccinated. In primary analysis of fully adjusted VE we included age and comorbidity as well as province and month for consistency with previous SPSN analyses [4] and those conducted elsewhere [10]. For reference we display analyses by individual covariate adjustment and also for age and comorbidity alone (Table 2).

**Table 2:** Vaccine effectiveness estimates against influenza A(H3N2), using any influenza virus or both influenza and SARS-CoV-2 virus test-negative controls, Canadian Sentinel Practitioner Surveillance Network, 6 March–2 July 2022 (n = 327)

Model	Any influenza virus test-negative controls		Both influenza and SARS-CoV-2 virus test-negative controls	
	n vac^a^/N	%	n vac^a^/N	%
Total	327		250	
Cases	15/42	36	15/42	36
Controls	155/285	54	108/208	52
Logistic regression models	VE %	95% CI	VE %	95% CI
**Unadjusted**	**53**	**9 to 76**	**49**	**−2 to 74**
**Univariate adjustment**				
For age group (1–8, 9–19, 20–49, 50–64, ≥ 65 years)	48	−10 to 75	43	−23 to 74
For province (AB, BC, ON)	51	3 to 75	43	−15 to 72
For comorbidity^b^	45	−11 to 72	38	−26 to 70
For calendar month (Mar, Apr, May, Jun–2 Jul)	50	1 to 75	45	−11 to 73
For age group and comorbidity^b^	46	−14 to 74	41	−29 to 73
**Full covariate adjustment (primary analysis)^c^**	**36**	**−38 to 71**	**24**	**−73 to 67**
Sensitivity analyses: Firth's penalised logistic regression	VE %	95% CI	VE %	95% CI
**Unadjusted**	**53**	**9 to 76**	**48**	**−2 to 74**
**Univariate adjustment**				
For age group (1–8, 9–19, 20–49, 50–64, ≥ 65 years)	46	−9 to 75	42	−23 to 73
For province (AB, BC, ON)	50	3 to 75	42	−14 to 71
For comorbidity^b^	44	−10 to 72	37	−26 to 70
For calendar month (Mar, Apr, May, Jun–2 Jul)	49	1 to 75	44	−11 to 72
For age group and comorbidity^b^	44	−14 to 74	39	−28 to 72
**Full covariate adjustment (as per primary analysis)^c^**	**35**	**−37 to 70**	**23**	**−71 to 66**

AB: Alberta; BC: British Columbia; CI: confidence interval; ON: Ontario; SARS-CoV-2: severe acute respiratory syndrome coronavirus 2; VE: vaccine effectiveness.

^a^ Vaccination status based on patients’ self-report; defined as receipt of 2021/22 seasonal influenza vaccine at least 2 weeks before symptom onset. Patients vaccinated less than 2 weeks before onset of symptoms or with unknown vaccination status or timing were excluded.

^b^ Includes chronic comorbidities that place individuals at higher risk of serious complications from influenza as defined by Canada’s National Advisory Committee on Immunization, including: heart, pulmonary (including asthma), renal, metabolic (such as diabetes), blood, cancer or immunocompromising conditions, conditions that compromise management of respiratory secretions and increase risk of aspiration, or morbid obesity (body mass index ≥ 40).

^c^ Age group (1–8, 9–19, 20–49, 50–64, ≥ 65 years), province (AB, BC, ON), comorbidity and calendar month (Mar, Apr, May, Jun–2 Jul).

Crude VE against influenza A(H3N2) was 53% (95% CI: 9–76). Adjustment for age and comorbidity had the greatest individual impacts in lowering VE estimates. In primary analysis, fully adjusted VE was 36% (95% CI: −38 to 71) (Table 2). Adjusted VE was lower, but with broadly overlapping confidence intervals, when excluding participants who were NAAT-positive or inconclusive for SARS-CoV-2 from the influenza test-negative controls (24%; 95% CI: −73 to 67). All VE estimates were similar when using Firth’s PLR to address sparse data (Table 2).

## Discussion

In the wake of COVID-19-related restrictions beginning March 2020, community-level influenza virus circulation essentially halted in Canada [1]. Two years later, as pandemic mitigation measures were relaxed, influenza A(H3N2) virus showed unusual late-season resurgence with a spring wave spanning March to July 2022 that abated to expected sporadic levels during the summer [2]. Despite mismatch of the vaccine clade 3C.2a1b.2a.1 strain against the circulating clade 3C.2a1b.2a.2 viruses and an unusually long time since vaccination, the Canadian SPSN shows that the 2021/22 vaccine reduced the risk of medically attended influenza A(H3N2) illness by about one third during the late spring wave. 

Confidence intervals around our VE estimates are wide and we cannot rule out an interpretation of no protection. However, point estimates remain the most likely findings and, despite a longer interval since vaccination, are comparable to VE estimates against influenza A(H3N2) recently reported from the United States (US) (35%; 95% CI: 19–47) for the period spanning October 2021 to April 2022 [10] and from Europe (35%; 95% CI: 6–54) spanning October 2021 to March 2022 [11]. Late-season findings of influenza VE for 2021/22 are within the range of prior SPSN estimates that are generally ≤ 50% against influenza A(H3N2) during the usual season from November to April, typically lower than against influenza A(H1N1) or influenza B [4], neither of which circulated in 2021/22 in Canada. As highlighted during the late-season 2018/19 influenza A(H3N2) epidemic that peaked in March 2019 and for which influenza A(H3N2) VE in Canada [12], the US [13] and Europe [14] was exceptionally low (< 20%), other factors beyond vaccine match or late-season waning, may also contribute to VE variation (e.g. immunological cohort effects) [12,14,15]. 

The current immuno-epidemiological context may be further complicated because circulation of influenza (and other respiratory viruses) virtually ceased during the pandemic, although insofar as that context was shared between vaccinated and unvaccinated individuals, it should not have influenced VE estimates. Excluding COVID-19 cases from the influenza virus test-negative controls lowered our influenza VE estimates (from about one third to one quarter), contrary to the direction of effect Doll et al. theorised due to positive collinearity between influenza and COVID-19 vaccination [3]. Underpinning that theory, in the context of effective COVID-19 vaccination, COVID-19 cases are less likely to be vaccinated against COVID-19, and if vaccine behaviours are correlated, then COVID-19 cases would also be less likely vaccinated against influenza. Excluding those who are less likely to be vaccinated against influenza from influenza test-negative controls would tend to increase influenza VE. However, among influenza virus test-negative controls in our data set, those who were COVID-19 cases were instead more often vaccinated against influenza and as such their exclusion may have tended instead to reduce influenza VE.

With respect to limitations, our results should be interpreted with caution given small sample size and wide confidence intervals. Residual bias and confounding cannot be ruled out. Generalisation to other jurisdictions where the mix of circulating viruses and other context differ should be undertaken cautiously.

## Conclusions

After a 2-year hiatus, influenza A(H3N2) viruses belonging to clade 3C.2a1b.2a.2 contributed to an atypical late spring 2022 wave in Canada. Despite an unusually long interval since vaccination, the mismatched 2021/22 vaccine reduced the risk of influenza A(H3N2) illness by about one-third, comparable to previous seasons. The findings reinforce the World Health Organization’s decision to switch to a more representative clade 3C.2a1b.2a.2 strain for the northern hemisphere 2022/23 A(H3N2) vaccine component. 

## Supplementary Data

177000 Supplement
