# Supplementary material for: Influenza vaccine effectiveness against A(H3N2) during the delayed 2021/22 epidemic in Canada
Source: Euro Surveill. 2022 Sep 22;27(38):2200720. doi: 10.2807/1560-7917.ES.2022.27.38.2200720 (PMC9511683; doi:10.2807/1560-7917.ES.2022.27.38.2200720)
Supplement: Supplement [file 22-00720_SKOWRONSKI_Supplement.pdf]

This supplementary material is hosted by *Eurosurveillance* as supporting information alongside the article "Influenza vaccine effectiveness against A(H3N2) during the delayed 2021/22 epidemic in Canada" on behalf of the authors, who remain responsible for the accuracy and appropriateness of the content. The same standards for ethics, copyright, attributions and permissions as for the article apply. Supplements are not edited by *Eurosurveillance* and the journal is not responsible for the maintenance of any links or email addresses herein.

Supplementary Table S1. Participant profile, including both influenza- and SARS-CoV-2 virus test-negative controls, 2021/22 influenza vaccine effectiveness evaluation, Canadian Sentinel Practitioner Surveillance Network (SPSN), 6 March 2022–2 July 2022 (n=250)

Characteristics	All participants (column %)							Proportion influenza vaccinated[a] (row %)						
	Overall		Influenza A(H3N2) cases		Both influenza- and SARS-CoV-2 virus test-negative controls[b]		p value[c]	Overall		p value[c]	Influenza A(H3N2) Cases		Both influenza- and SARS-CoV-2 virus test-negative controls[b]	
	n	%	n	%	n	%		n	%		n	%	n	%
**N (row %)**	250	100	42	17	208	83	NA	123	49	NA	15	36	108	52
**Age group (years)**														
1-8	58	23	6	14	52	25	0.002	18	31	<0.001	2	33	16	31
9-19	42	17	16	38	26	13		14	33		3	19	11	42
20-49	86	34	13	31	73	35		37	43		5	38	32	44
50-64	33	13	3	7	30	14		26	79		1	33	25	83
65+	31	12	4	10	27	13		28	90		4	100	24	89
**Median (range)**	29 (1-95)		18.5 (2-81)		30.5 (1-95)		0.292	43 (1-95)		<0.001	38 (2-81)		43 (1-95)	
**Interquartile range**	10-50		13-38		8.5-54.5		NA	19-63		NA	18-66		19.5-62.5	
**Sex**														
Female	148	59	22	52	126	61	0.290	77	52	0.278	9	41	68	54
Male	100	40	20	48	80	38		45	45		6	30	39	49
Unknown	2	1	0	0	2	1	NA	1	50	NA	0	0	1	50
**Comorbidity[d]**														
No	182	73	36	86	146	70	0.039	72	40	<0.001	11	31	61	42
Yes	68	27	6	14	62	30		51	75		4	67	47	76
**Province**														
Alberta	106	42	25	60	81	39	0.042	44	42	0.049	6	24	38	47
BC	58	23	8	19	50	24		28	48		2	25	26	52
Ontario	86	34	9	21	77	37		51	59		7	78	44	57
**Specimen collection interval from onset of influenza-like illness (ILI)[e]**														
≤4 days	180	72	35	83	145	70	0.073	90	50	0.685	12	34	78	54
5-7 days	70	28	7	17	63	30		33	47		3	43	30	48
**Median**	3		2.5		3		0.013	3		0.368	3		3	
**Month of specimen collection, 2022**														
March	48	19	4	10	44	21	0.066	26	54	0.154	1	25	25	57
April	78	31	18	43	60	29		30	38		4	22	26	43
May	73	29	15	36	58	28		39	53		8	53	31	53
June-July 2nd	51	20	5	12	46	22		28	55		2	40	26	57

NA: not applicable.

Unless otherwise specified, values displayed in columns represent the number of specimens per category and percentages are relative to the total.

[a] Vaccination status based on patients' self-report; defined as receipt of 2021/22 seasonal influenza vaccine at least 2 weeks before symptom onset. Patients vaccinated less than 2 weeks before onset of symptoms or with unknown vaccination status or timing were excluded.

[b] Excludes 77 influenza virus test-negative control specimens that tested positive (n=76) or inconclusive (n=1) for SARS-CoV-2 virus by nucleic acid amplification test (NAAT). As such, controls displayed here include those that are both influenza and SARS-CoV-2 virus test-negative.

[c] p values for comparison between cases and controls or for the proportion vaccinated were derived by chi-squared test or Wilcoxon rank-sum test.

[d] Includes chronic comorbidities that place individuals at higher risk of serious complications from influenza as defined by Canada's National Advisory Committee on Immunization, including: heart, pulmonary (including asthma), renal, metabolic (such as diabetes), blood, cancer or immunocompromising conditions, conditions that compromise management of respiratory secretions and increase risk of aspiration, or morbid obesity (body mass index ≥ 40).

[e] As per usual SPSN approach, missing specimen collection dates were imputed as the date the specimen was received and processed at the laboratory minus 2 days.
